# Development and Implementation of an Ultraviolet-Dye-Based Qualification Procedure for Hand Washing and Disinfection to Improve Quality Assurance of Pharmacy Preparations and Compounding, Especially in Cleanrooms: A Pilot Study

**DOI:** 10.3390/pharmacy12030073

**Published:** 2024-04-25

**Authors:** Catharina W. J. Knol, Paul H. Stob, Herman J. Woerdenbag

**Affiliations:** 1 Department of Pharmaceutical Technology and Biopharmacy, Groningen Research Institute of Pharmacy (GRIP), University of Groningen, Antonius Deusinglaan 1, 9713 AV Groningen, The Netherlands; c.w.j.knol@gmail.com; 2 Fagron Sterile Services Nederland, Dieselstraat 3, 7903 AR Hoogeveen, The Netherlands; p.h.stob@gmail.com

**Keywords:** aseptic handling, cleanroom personnel, contamination control, extemporaneous compounding, Good Manufacturing Practice (GMP), hand washing and disinfection, hygiene training, pharmacy preparations, sterile manufacturing, UV-dye-based assessment

## Abstract

Even though, nowadays, most medicines are manufactured industrially, patients may have medical needs that can only be met by a tailor-made approach. This requires the availability of pharmacy preparations made under Good Manufacturing Practice (GMP) conditions. An efficient hand hygiene practice is essential herewith, especially if sterile products that are prepared in a cleanroom are concerned. The effectiveness of hand washing and hand disinfection procedures greatly relies on adequate training. We carried out an observational cross-sectional pilot study aimed at optimizing hand hygiene training with objective and measurable quality assessments using an ultraviolet (UV) dye. Practical acceptance criteria for qualifying personnel through this method were set and evaluated. In total, 25 GMP-qualified cleanroom operators washed and disinfected their hands with UV dye hand wash lotion and UV dye hand alcohol, respectively. To obtain a proof-of-concept, the results were judged based on adherence to the WHO six-step protocol and associated acceptance criteria. Commonly missed areas were brought to light, and the influence of procedure duration was investigated. UV-dye-based assessments appeared to be more valuable in hand disinfection than in hand washing. In both procedures, the back of the hands and the thumbs were frequently missed. This underpins the need for enhanced and repeated education on hand washing and disinfection. Additionally, a dry skin gave rise to extra cleaning challenges. From this pharmacy practice pilot study with a focus on pharmaceutical product care, it may be concluded that the application of UV-dye-based assessments offers valuable insights for pharmacists to optimize hand hygiene, thereby increasing the safety of tailor-made medicines and on-site preparations.

## 1. Introduction

It is the duty of pharmacists to dispense medicines that best fit a patient’s medical needs [1,2,3]. Historically, pharmacists used to make medicines themselves, but, nowadays, the majority of the medicines are manufactured industrially [4]. While a one-size-fits-all approach with commercially available formulations works for many patients, it is definitely not suitable for all. Particularly for children, a tailor-made adjustment to the dose or dosage form may be desired to meet the specific needs in clinical practice [5,6]. Furthermore, alternative solutions are sought if a patient is best treated with a medicine unavailable as a commercial formulation [2,4,7] or to cope with the worldwide recurring supply shortages [5,8].

In all these cases, pharmacy preparations, which are also named extemporaneous preparations or compounded products, are in place. Pharmacy preparations are currently undergoing a revival after being considered less popular and declined for reasons of poor quality in the recent past [9]. The introduction of advanced therapy medicinal products (ATMPs) requiring on-site modifications also emphasizes the importance of having pharmacists available who possess the necessary knowledge, skills, and competences for these practices, combined with ample experience in quality assurance and quality control [2,5]. The increased demand for tailor-made medicines has led to the development of large-scale compounding pharmacies with specialized facilities and equipment in many countries, where pharmacists prepare and distribute significant quantities of commercially unavailable medications to patients, hospitals, and other (non-compounding) pharmacies [4].

According to the Hepler and Strand definition of pharmaceutical care, medicines are administered for the purpose of achieving definite outcomes that improve the patient’s quality of life [10]. This cannot be achieved without having access to qualitatively good and safe products. This applies to industrially manufactured medicinal products as well as to tailor-made medicines and on-site modifications that fall under the responsibility of pharmacists.

Pharmacists play an increasingly important role in the on-site preparation of injections to make them ready for administration in order to reduce medication errors and improve patient safety [5]. Most hospital pharmacies maintain aseptic compounding facilities with cleanrooms to be able to provide their patients with the required individual dosing and with formulations that are not available from a commercial source [7]. There has been a clear shift from carrying out activities related to parenteral dosage forms at the ward of hospitals under limited product protection to cleanrooms where dedicated and appropriately trained staff prepare the required products under enhanced or maximal product protection using aseptic techniques [11,12]. This approach has been shown to mitigate risks in aseptic preparations and reconstituted parenteral medicines in healthcare establishments, contributing to the improvement of patient safety and the production of optimal aseptic products [12,13,14]. This sterile compounding practice is recognized as essential for pharmaceutical care for the benefit of patients and health services [15].

As the responsibility of tailor-made medicine production lies with pharmacists, it is their task to optimize the practice of pharmacy preparations and monitor the environment, equipment, and procedures, ensuring that the highest quality is obtained and sterility maintained [7]. That a failure in this respect can be catastrophic is illustrated by a well-known case from 2012 in the Unites States. Poor microbiological quality of methylprednisolone acetate injections from a compounding pharmacy resulted in 137 cases of *Aspergillus fumigaticus* and *Exserohilum rostratum* fungal meningitis and 12 deaths [16,17]. Over time, many more medication errors associated with injectable medicines, severe harm, and deaths have been reported [4,18]. In response to these serious events linked to the poor quality of compounded medicines, the Drug Quality and Security Act was published in 2013, giving the FDA more authority to regulate and monitor manufacturers of compounded products [19,20].

Nowadays, non-sterile and sterile pharmacy preparations, recognized as a fundamental part of pharmacy practice, are subject to strict international quality standards which are legally binding. Compounding pharmacists are obliged to follow them in order to produce safe and effective personal medication [21]. To get a better hold on the preparation of medicines in pharmacies, the European Pharmacopoeia (Ph. Eur. 11) contains the monograph *Pharmaceutical Preparations* [22]. The guidelines of the American Society of Health-System Pharmacists (ASHP) [23] are included in the United States Pharmacopeia (USP) monograph <797> *Pharmaceutical Compounding—Sterile Preparations* [24]. With respect to pharmacy preparations, the USP also contains monograph <795> *Pharmaceutical Compounding—Non-Sterile Preparations* [25] and monograph <1075> *Good Compounding Practices* [26].

To achieve a robust quality of pharmaceutical preparation, adherence to the Good Manufacturing Practice (GMP) guidelines is globally supported. These guidelines should guarantee the proper application and validation of procedures as well as the quality and safety of pharmaceutical preparations [27,28,29,30]. Full compliance with the GMP guidelines is compulsory in both the European Union and the Unites States for outsourcing facilities [1,19]. The GMP guidelines are especially applicable to high-risk preparations. For low-risk preparations, the PIC/S GPP guidelines [31] are often used as a reference for appropriate quality systems. However, it has been shown that compounding practices for pediatrics that are widely applied in developing countries often lack adherence to these quality guidelines. An important risk factor is a lack of awareness, knowledge, and competence among compounding staff [32].

One of the essential elements of the GMP guidelines (mentioned in GMP Chapter 1) is that Pharmaceutical Quality Systems should facilitate innovation and continual improvement [9]. Quality assurance aspects of the GMP, described in detail in the (recently revised) Annex 1 [33], include the hygiene of compounding personnel. This is crucial for preventing microbial contamination of products, especially those for parenteral administration [34]. Therefore, a globally applied pharmacopoeia requirement is that parenteral dosage forms are sterile [35].

Important in ensuring the quality of sterile medicinal products is the use of cleanrooms, in which microbiological contamination risks are minimized. One of the significant contributors to microbial contamination in cleanrooms are the operators [34]. According to the EU directive 2003/94/EC Chapter 7 (Personnel), it is mandatory to establish and maintain a hygiene program for personnel working in cleanrooms [30]. Also, USP monograph <797> contains numerous requirements in relation to this topic [24]. An essential part of the hygiene program includes hand washing and disinfection procedures. People have been cleaning their hands with water and soap for centuries. Originally, this practice had aesthetic or religious reasons. Following the observation by scientist Ignaz Semmelweis in 1847 that a correlation existed between maternal mortality and the way doctors cleaned their hands, hand hygiene has also become medically relevant. Hand hygiene has ever since been recognized as the most effective method to prevent the transmission of microorganisms and pathogens [36].

While hand washing is intended to remove particles and visible dirt, disinfection has been proven to be the most effective method for combating the contamination of materials with microorganisms, according to the extensive literature review conducted by the WHO [37]. For both practices, the application technique is essential for achieving optimal results.

**Figure 1:**
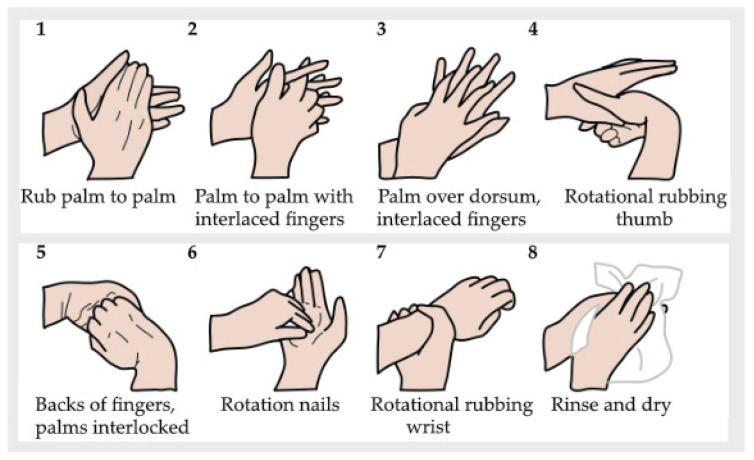
Hand rubbing motions in hand washing and hand disinfection, based on the WHO six-step protocol [39].

Annex 1 of the GMP guidelines which focuses on sterile preparations demands written protocols for hand hygiene procedures designed to minimize transfer of contaminants to clear areas [33]. To standardize these, the World Health Organization (WHO) in 2009 published the *WHO guidelines on hand hygiene in health care* [37]. These guidelines recommend a six-step protocol that was originally developed by the British medical biologist Graham Ayliffe in 1978 [38]. A frequent addition to this WHO protocol is the inclusion of the wrists [39], as depicted in Figure 1. Antiseptic solution ‘EPG’, containing ethanol, hydrogen peroxide, and glycerin, has been recommended by the WHO to be used for both hand hygiene and antisepsis [40].

Training and qualification for compounding practices require operators to demonstrate proficiency in following both hand washing and hand disinfection protocols. Furthermore, according to the *WHO best practices for injections and related procedures toolkit*, hand hygiene should be performed prior to the onsite preparation of injection materials, that is, before entering a cleanroom [41].

Proficiency is typically assessed through direct observation of the actions performed. However, this method solely examines the execution of the hand rubbing motions, while neglecting to validate the quality of those actions. While the effectiveness of the six-step actions in reducing bacterial load has been demonstrated in a systematic review by Price et al., differences in contact time, technique execution, and applied volume of soap or disinfectant can lead to variations in outcome [42]. Therefore, expanding hand hygiene training with an objective assessment method focusing on the quality of technique may offer potential benefits.

The conventional way to assess the quality of hand hygiene is by microbiological sampling, offering a direct measure of microbial contamination of the hands in the form of colony-forming units (CFUs) on an agar Petri dish. However, this method is time-consuming, as it typically requires incubation periods of 48 h or longer before the result can be read out [43]. Because of this, cleanroom operators are not able to get immediate feedback on their performance, limiting the training potential. Furthermore, the sampling as such will pose challenges. Conventional Petri dishes are too small to capture a hand’s entire surface area, resulting in indirect measurements through glove imprints [43,44] or limited representation of the selected fingers only [38,45,46]. Additionally, there is considerable variation in initial contamination levels, making it difficult to determine the actual reduction factor achieved as an estimate of the effectiveness of the procedure applied.

An ultraviolet (UV)-dye-based assessment method offers a faster and less labor-intensive alternative to microbiological control, as it can be performed easily and the result can be read out within a few minutes. Moreover, it offers operators immediate feedback on their performance. A UV-dye-based assessment is an indirect fluorescence measurement method based on the principle that if the entire hand surface is subjected to washing and disinfection, it should be free from microorganisms and particles. This concept was tested by Lehotsky et al. and validated against the microbiological assay. A UV-dye-based assessment was demonstrated to yield high sensitivity (95%) and specificity (98%) [47]. However, for implementing this method as a routine qualification procedure for hand hygiene, specific criteria must be investigated to obtain a proof-of-concept. In the GMP guidelines [27,28,29], requirements are formulated, but no detailed procedures are described with respect to how to assess them. By having a proof-of-concept, a UV-dye-based assessment may become part of in-house training programs for cleanroom operators.

The current pilot study aimed to optimize hand hygiene training by objectively assessing the quality of hand hygiene procedures using a UV-dye-based method in a limited-size group of cleanroom operators. Practical acceptance criteria for qualifying personnel through this method were set and evaluated. In addition, we aimed to identify commonly missed hand areas and investigated the potential impact of procedure duration.

## 2. Materials and Methods

### 2.1. Study Design, Location, and Participants

This observational cross-sectional pilot study was conducted in March–April 2023 with cleanroom operators working at Fagron Sterile Services Nederland (Hoogeveen, The Netherlands). The group consisted of 25 operators. Of them, 21 participated in the hand washing procedure, while all 25 participated in the hand disinfection procedure. The group (one male, 24 females; age 20–60 years) encompassed the majority of the cleanroom workers employed at Fagron Sterile Services and was considered to be representative. Most of them were educated as pharmacy technicians. All had previously been qualified according to GMP for hand hygiene practices for sterile pharmacy preparations through direct observation. In The Netherlands, a GMP qualification is required for all compounding activities by reselling pharmacies. To fully anonymize personal data, each cleanroom operator in the study was identified with a number. All cleanroom operators were informed about the aim of the study and consented to participate.

### 2.2. Hand Washing

**Figure 2:**
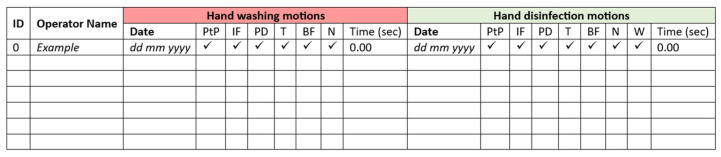
Example template of the form used to record the performed actions in this research. Abbreviations: ID = unique identification number of the cleanroom operator; PtP = rub palm to palm; IF = palm to palm with interlaced fingers; PD = palm over dorsum interlaced fingers; T = rotational rubbing thumb; BF = backs of fingers palms interlocked; N = rotation nails, W = rotational rubbing wrist. See Figure 1 for the illustration of each motion.

Before hand washing, the operators applied 2–3 mL of UV dye hand washing lotion (two-to-three pumps) (Allergenen Consultancy BV, Scherpenzeel, The Netherlands), which was spread carefully to wet the entirety of both hands. Subsequently, the hands were checked under an ultraviolet lamp (Philips TL 8w BLB) installed in an ambient-light-reducing box (Derma LiteCheck^®^ Box, Paul Hartmann BV, Heidenheim, Germany) to ensure that the fluorescence light covered the entirety of both hands. After this had been confirmed, the operators performed the hand washing steps according to the WHO six-step protocol, as shown in Figure 1. Around 3 mL of hand washing lotion (Baktolin^®^ pure, Paul Hartmann BV) was diluted with tap water (the volume depended on the washing time). This is compliant with the WHO protocol for hand hygiene [39]. The performed actions were monitored by the researcher (C.W.J.K.)**,** and the total duration of the hand washing procedure was recorded. The results were registered on a dedicated form (Figure 2).

**Figure 3:**
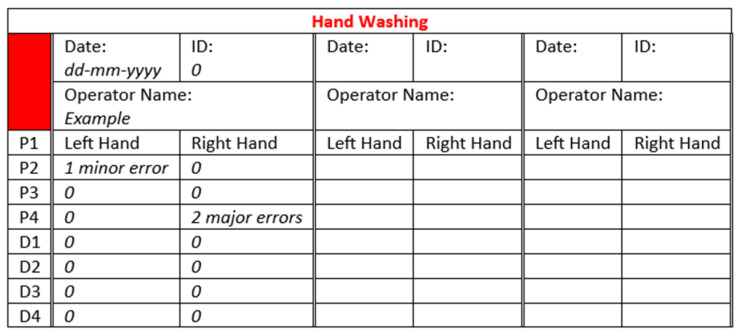
Example template of the form used for hand washing to record analysis of the pictures with errors per hand area. Abbreviations: ID = unique identification number of the cleanroom operator; P = palmar; D = dorsal. P and D refer to the division into hand areas, as shown in Figure 4.

Subsequently, it was checked in the Derma LiteCheck^®^ Box whether all parts of the hands had lost the fluorescence by taking and evaluating a photograph (8 MP Camera, Xiaomi REDMI A1, Beijing, China). The results were registered manually on a form (Figure 3).

**Figure 4:**
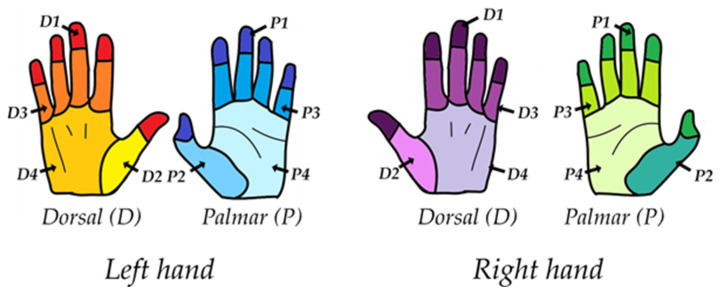
Region division used in the study, based on the method by Vanyolos et al. [48]. D1: fingertips and distal phalanges dorsal side of the left hand (red) and right hand (purple). P1: fingertips and distal phalanges palmar side of the left hand (blue) and right hand (green). D2: the thumb and the first metacarpal bone of the dorsal side of the left hand (yellow) and right hand (pink). P2: the thumb and the first metacarpal bone of the palmar side of the left hand (blue) and right hand (green). D3: the middle and proximal phalanges of the dorsal side of the left hand (orange) and right hand (purple). P3: the middle and proximal phalanges of the palmar side of the left hand (blue) and right hand (green). D4: the second-to-fifth metacarpal bones and the main hand surface dorsal side of the left hand (dark yellow) and right hand (light purple). P4: the second-to-fifth metacarpal bones and the main hand surface of the palmar side of the left hand (light blue) and right hand (light green).

### 2.3. Hand Disinfection

**Figure 5:**
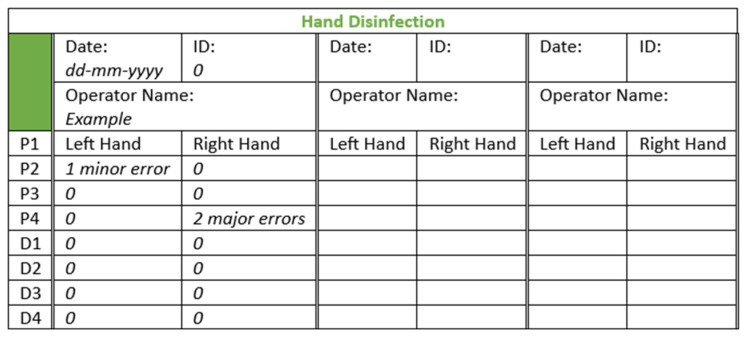
Example template of the form used for hand disinfection to record analysis of the pictures with errors per hand area. Abbreviations: ID = unique identification number of the cleanroom operator; P = palmar; D = dorsal. P and D refer to the division into hand areas, as shown in Figure 4.

Prior to performing the hand disinfection procedure, the hands were checked under the UV lamp to ensure there was no background fluorescence due to a possible residue from the hand washing procedure. Disinfection was performed by following the prescribed hand hygiene steps according to the WHO six-step protocol, as shown in Figure 1, using 2–3 mL of an ultraviolet-colored disinfectant solution (two-to-three pumps) (Visirub, Paul Hartmann BV + SkinmanTM Soft Protect, Ecolab, Hamburg, Germany). The final volume was that needed to fully wet the hands and wrists (compliant with the WHO protocol for hand hygiene [39]). If the solution dried up before the entire procedure was finished, a second layer of hand disinfection solution (1–3 mL) was applied. The performed actions were monitored, and the total duration of the hand disinfection procedure was recorded. The results were registered on a form (see Figure 2). Afterwards, it was checked in the Derma LiteCheck^®^ Box whether all parts of the hand were fluorescent by taking and evaluating a photograph (8 MP Camera, Xiaomi REDMI A1, Beijing, China). The results of this were registered manually on a form (Figure 5).

### 2.4. Analysis of Photographic Material

The surface area of the hands was visually assessed by including a square of 0.6 cm^2^ in the photographs. A missed area larger than 0.6 cm^2^ was defined as a major error, while a missed area smaller than 0.6 cm^2^ was defined as a minor error [49]. The threshold to qualify for a successfully executed hand washing or hand disinfection procedure was set to a maximum of two minor errors, consistent with the criteria of Szilagyi et al. [49]. No major errors were allowed. To determine the distribution of possible errors over the hand areas, the method of Vanyolos et al. was used [48], which divides the palm and back of the hand into four regions each (see Figure 4).

Region 1 includes the fingertips and the distal phalanges. Region 2 includes the thumb and the first metacarpal bone. Region 3 includes the middle and proximal phalanges. Region 4 includes the second-to-fifth metacarpal bones and the main hand surface. With respect to each region, the number of errors was counted for each cleanroom operator. The left and right hands were counted separately alongside the palm and the back sections of each hand. The results were registered manually on the form used to monitor hand washing and hand disinfection procedures (see Figure 3 and Figure 5).

### 2.5. Statistics

To determine the possible statistical difference between the number of qualified operators before and after the introduction of the UV-dye-based assessment method, a McNemar test (two-tailed) was performed in SPSS, with the variables defined as ‘all actions performed’ (yes/no) and ‘in agreement according to the UV-dye-based assessment’ (yes/no). A *p*-value < 0.05 was considered significant.

## 3. Results

### 3.1. Adherence to Hand Washing and Hand Disinfection Procedures

In Table 1, the adherence by the cleanroom operators to the WHO six-step protocol for hand washing and hand disinfection is shown. All 21 cleanroom operators performed all prescribed hand washing actions, with 90% meeting the requirement set for the UV-dye-based assessment. There was no significant difference (*p* = 0.05) between the results of the UV-dye-based assessment and the observational assessment (WHO six-step protocol adherence). All 25 cleanroom operators performed all prescribed hand disinfection actions, with 76% meeting the requirement set for the UV-dye-based assessment. There was a significant difference (*p* = 0.03) between the results of the UV-dye-based assessment and the observational assessment (WHO six-step protocol adherence).

### 3.2. Missed Hand Areas in Hand Washing and Hand Disinfection Procedures

In Table 2, errors made by the cleanroom operators in hand washing and hand disinfection are shown. Using the UV-dye-based assessment to evaluate the hand washing procedure, errors were found with respect to three cleanroom operators. Two of them did not meet the requirement, with one accumulating 16 minor errors relative to each hand area and the other accumulating four major errors with respect to the back of the hand.

**Figure 6:**
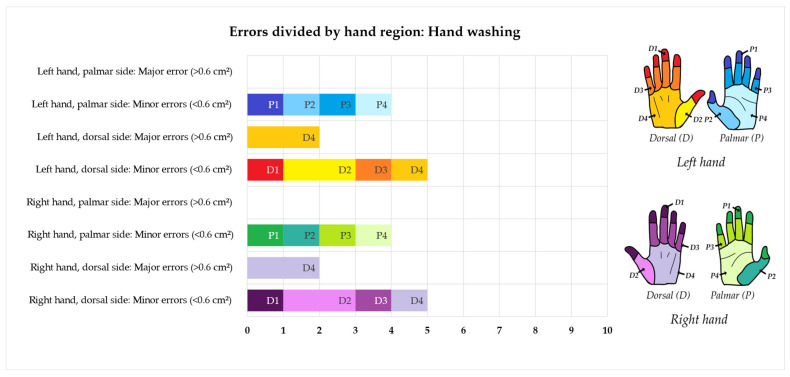
Minor and major errors in the hand washing protocol divided by hand region. The dorsal side of the left hand is depicted in red/orange, the palmar side of the left hand is depicted in blue, the dorsal side of the right hand is depicted in purple, and the palmar side of the right hand is depicted in green. Errors were located using the UV-dye-based assessment method.

**Figure 7:**
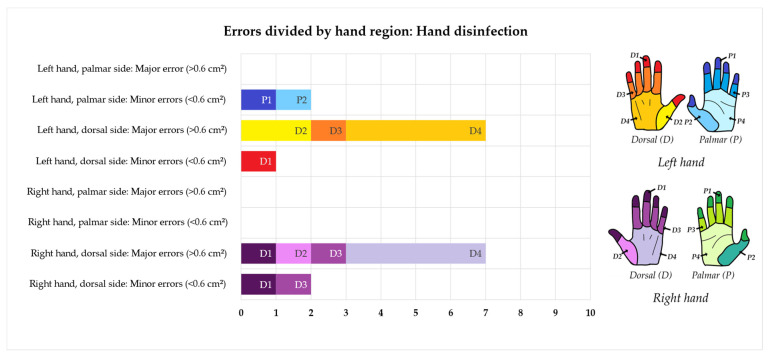
Minor and major errors in the hand disinfection protocol divided by hand region. The dorsal side of the left hand is depicted in red/orange, the palmar side of the left hand is depicted in blue, the dorsal side of the right hand is depicted in purple, and the palmar side of the right hand is depicted in green. Errors were located using the UV-dye-based assessment method.

As it can be seen in Figure 6, the majority of errors in hand washing were made at the thumb creases and at the back of the hands. There was no difference between the left and right hands. Using the UV-dye-based assessment to evaluate the hand disinfection procedure, errors were found on eight cleanroom operators. Six of them committed one or more major errors, while two committed one minor error each. In total, five minor errors and 14 major errors were found. As it can be seen in Figure 7, most of the errors in disinfection were detected in relation to the back of the hands. The creases between the fingers, the fingertips, and the thumb creases on the back of the hands were also frequently missed. There was a minor difference between the right and left hands, with ten errors found on the left and nine errors found on the right hand.

**Figure 8:**
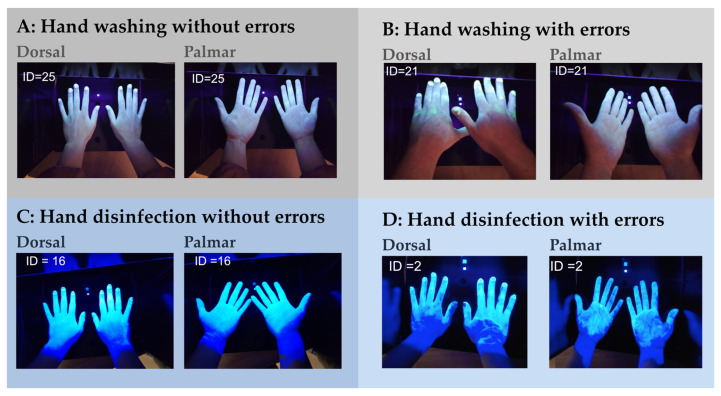
Representative examples of photographs of residual fluorescence after hand washing ((**A**): without errors, (**B**): with errors) and after hand disinfection ((**C**): without errors, (**D**): with errors).

Figure 8 shows representative examples of photographs of residual fluorescence after hand washing and after hand disinfection.

### 3.3. Procedure Duration: Influence of Time on Efficacy

The average time required for hand washing when the efficacy was found to meet the requirements was 101.6 s (median value 98.9 s) (see Table 3). The standard deviation and interquartile range of these performances were high, indicating a relatively large variation in the recorded times. For performances that did not meet the requirements for hand washing, the average duration was slightly longer, but concerned only two cases.

The average time required for hand disinfection when the performances met the requirements was 67.9 s (median value 65.8 s). Again, in these measurements, a considerable variation was observed, resulting in a high standard deviation. The average time required for hand disinfection when requirements were not met was nearly identical. The standard deviation and interquartile range, however, were considerably lower, meaning that the recorded values were in closer proximity to one another. Note that the procedure time was not included in relation to all participants, as some had already started washing before the timer was set. In other cases, the timer was stooped too late. This led to five inaccurate values that were excluded from the analysis.

## 4. Discussion

This pharmacy-practice-related pilot study focuses on pharmaceutical product care, with the objective of making qualitatively better and safer sterile products available to the patients in support of pharmaceutical patient care. It provides a practice to upgrade hand hygiene procedures to work in cleanrooms by which an applicable pharmaceutical quality management system can be innovated and improved, as demanded in Chapter 1 of the GMP [27].

The cleanroom operators in our study all showed complete adherence to the WHO six-step protocol. The pass rate of the UV-dye-based assessment was lower, 90% for hand washing and 76% for disinfection. Nevertheless, this is still quite high in comparison to earlier studies. In research conducted by Szilágyi and coworkers on disinfection, 72% of the 4642 hospital cleanroom operators included in that study met the criterion when using the same procedures after having received instructions [49]. Another study using surface coverage, conducted by Ványolós et al., found a pass rate of 51% in 253 medical students after a first instruction, such a rate increasing to 74% after a second instruction [48]. Those two studies were conducted with unexperienced subjects, while our cleanroom operators had a more extensive experience with hand hygiene protocols prior to the study. This is a plausible explanation for the higher pass ratio in our test group. Moreover, Lehotsky et al. showed that the pass rate tends to improve with more frequent feedback [50]. This clearly indicates that experience can enhance hand hygiene performance.

Despite the found compliance in our study with the WHO six-step protocol for both the hand washing and disinfection procedures, there were also cleanroom operators who failed to meet the requirements set for the UV-based assessment method. Particularly, when it came to hand disinfection, a significant number of cleanroom operators did not meet the Szilágyi criteria [49]. The observed disparity between hand washing and disinfection may be explained by several factors. One explanation lies in understanding the importance of the hand rubbing motions. Their significance in the hand washing protocol was evident to all participants, but, in the context of disinfection, it remained less apparent. This highlights the need for enhanced and repeated training to increase and maintain awareness and ensure better compliance, especially in hand disinfection. Another explanation suggests an important role of the volume of water applied. In hand washing, there is a continuous flow of moving water and soap foam, making the rubbing hand motions less crucial than when applying a ‘static’ volume of 3 mL of disinfection solution, as done during hand disinfection [51].

Another finding emphasizing the need for improved training was that the most common errors in both hand washing and hand disinfection were found in relation to the back of the hands and thumbs. This is consistent with the research by Lehotsky et al., where thumbs and back were identified as commonly missed [47]. In the systemic review by Wong et al., the back of the hands was listed among the top three of missed areas. The feature of missed areas can be reduced by avoiding excessive palm curvature during motions covering the back of the hands and by paying extra attention to the thumb area [52]. In cases were parts of the hands of operators in our study were still fluorescent, the operators were asked to repeat all handlings according to the instructions while feedback was given to improve attention to the insufficiently treated parts.

Next to training purposes, the UV-dye-based assessment method can be implemented as a way of qualification using the Szilágyi criteria [49]. Marking the treated surface with UV dye provides a reliable indication of the reduction in microbiological burden. The hand alcohol and hand washing lotion comply with European standards EN 1500 and EN 14885, providing evidence of virus and bacteria reduction on sufficiently treated surfaces that in the assessment are made visible with the UV dye [53]. A correlation between UV dye visibility and reduction of contamination of treated surface areas has been demonstrated by Turner et al. [54] for hand washing assessments and has been validated by Lehotsky et al. [47] in the context of hand disinfection using a UV dye. However, despite the established link between the UV dye covered surface and the reduction of contamination, a quantitative determination of the reduction factor according to the Szilágyi criteria is yet to be established [49]. Additionally, interpretation by different observers and further investigation of the effect of hand hygiene of personnel on the safety and stability of produced medicines may provide insightful revelations [55]. Nevertheless, when the average hand surface area of women (392 cm^2^) and men (448 cm^2^) is considered, even with the two small errors allowed according to the Szilágyi criteria [49], 99.70% and 99.74% of the hand surface area were covered by the applied product [56], respectively. Also, when considering alternatives, such as calculating the surface percentage, the Szilágyi criteria [49] remain the most favorable option for qualification assessments. Other UV-dye-based assessment methods typically require the use of advanced photo analysis software and are highly reliant on the camera resolution utilized [41,47,49].

An interesting observation was made during hand washing. For one cleanroom operator, the UV dye was still visible on the skin folds after more than 2.5 min of hand washing and performing the set of hand washing motions twice. Unlike the other cleanroom operators, this person had a very dry and aged skin. This is an important finding, since cleanroom operators are likely to encounter dry hands frequently because of the intense hand hygiene practices to which they are continuously exposed. The visibility of UV dye after multiple washing attempts suggests that UV coloring may accumulate on the skin surface in the case of dry or aged skin. Although the risk of systemic absorption of substances is generally lower when applied to a dry skin compared to a hydrated skin, deeper folds can trap a substance on the skin surface [57]. This may yield false positive results when using the UV dye method as a way of qualification. To rule out such false positives, the fold area can be considered to lie within the error margin, as a skin fold has an area lower than 0.6 cm^2^. Alternatively, the skin can be pretreated with a moisturizer such as petroleum jelly up to half an hour before the training starts to assess the outcome based on a hydrated skin.

The average procedure duration in our study exceeded the WHO recommended time of 1 min for hand washing and 20–30 s for hand disinfection [37]. No clear correlation was observed between the UV-dye-based assessment outcome and the measured time to complete the procedure. Regarding hand washing, a time–effectiveness relationship of 5 s to 30 s has been established by Deochand et al. [58]. However, regarding disinfection, it is more complex: Kampf et al. concluded that hand rubbing motions are more relevant for the quality of the procedure than time, although disinfection shorter than 15 s was not found to be sufficient [59]. The measured times in our study were considerably longer than those reported in the literature, possibly because the cleanroom operators were very experienced with the protocol up front or due to a detection bias.

Another factor influencing the effectiveness of hand disinfection is the volume of disinfectant solution applied. It was found in our study that, after the 4th step (see Figure 1) of the disinfection process, the majority of the operators required a second portion of 1–3 mL hand alcohol as the prescribed actions could not be completed within the drying time. This seems to depend on technique and probably experience, as some operators did achieve complete coverage within 20–30 s. Research by Nemeth et al. also showed that increasing the volume of hand alcohol from 1.5 mL to 3 mL is effective only when the actions are performed correctly [51]. Increasing the volume of hand alcohol can provide operators with the extra time needed to complete the actions, even with a slower hand movement speed. Therefore, our recommendation would be to apply a second volume whenever hands get dry during the execution of the disinfection protocol. Furthermore, it may be interesting to investigate the minimal volume of hand alcohol needed to achieve the same coverage as with the hand washing solution. In this context, the sensitivity of the UV detection method in relation to the volumes needed has to be taken into account to confirm disinfection with other methods, as the sensitivity of detection depends on the applied volume.

The design of our observational cross-sectional pilot study has several limitations. The number of included participants was relatively small and lacked a control group. Multiple observations per participant would be a sound extension for a broader designed study. The potential for the Hawthorne effect, the increased awareness of being monitored, possibly impacting behavior and handling [60], cannot be ruled out, although cleanroom operators are used to being supervised during their work. Only one compounding center was included. An observational bias cannot be excluded. No discrimination was made between dominant and non-dominant hands. Years of working experience in cleanrooms and age of the operators were not included as variables, possibly influencing the outcome of our pilot study. All operators were well-trained and GMP-qualified to work in a cleanroom, a fact that is probably more relevant than age or year of experience.

Another limitation of our pilot study is a lack of microbiological data. Future research may therefore be directed toward the identification of microorganisms present on the hands before and after the application of the UV dye hand wash lotion and the UV dye hand alcohol. The results of such work are needed for the thorough validation of the hand washing and hand disinfection processes. Molecular techniques are the current standards to identify microorganism species, having replaced the classical biochemical assays (phenotypic methods) [61,62]. DNA sequencing of the 16S ribosomal RNA (rRNA) (part of the 30S subunit of the prokaryotic ribosome) is used to identify bacteria, while the 18S rRNA (part of the 40S subunit of the eukaryotic ribosome) is used to identify fungi [63]. A new development in the identification of microorganisms is the use of matrix-assisted laser desorption-ionization time-of-flight (MALDI-TOF) mass spectrometry (MS) [64].

## 5. Conclusions

Taken altogether, this study shows that the UV-dye-based assessment criteria of Szilágyi and coworkers [49] can be successfully applied to training and qualification of hand hygiene of cleanroom operators as required in the GMP guidelines. By using the UV-dye-based assessment method, errors that cannot be detected through simple observation of compliance using the WHO six-step protocol can be identified. It thereby uncovers opportunities to enhance hand hygiene training, especially for disinfection purposes, but also for showing commonly missed areas such as the back of the hands and the thumbs. The method may be used for the training of newly appointed cleanroom workers as well as for the regular assessment of GMP-qualified personnel.

The costs involved are limited and estimated to be around € 1000 at most for the UV lamp and the ambient-light-reducing box. However, writing the protocol and the instructions requires an investment of time. In addition to these one-time investments, there will be returning costs for the washing lotion and disinfection solution depending on the number of operators.

An important advantage compared to the application of microbiological techniques is the immediate visibility of the result. This makes it very useful and illustrative for educational purposes. When implementing the UV-dye-based method, it is recommended that dry and aged skin is hydrated in advance to avoid false positives. Moreover, the volume of applied hand disinfection solution should sufficiently wet the hands and keep the hands wet during the execution of all hand rubbing motions. Challenges for further improvement include the validation of several elements of the study design (e.g., the volume of hand washing and hand disinfection lotion applied and the size of the square used to visually assess the hand surface area). Further investigations into the microbiological and particle reduction factor in relation to the criteria could provide additional evidence to support the UV-dye-based assessment of hand hygiene practices. Finally, a deepening of our pilot study can be achieved by carrying out a microbiological analysis, applying molecular techniques.

## Figures and Tables

**Table 1:** Results of two investigated qualification methods for hand washing: observation of the execution of actions (WHO six-step protocol) and the UV-dye-based assessment. The numbers indicate the respective number of cleanroom operators.

	Hand Washing			Hand Disinfection		
Actions Correctly Executed	Met UV Requirement	Did Not Meet UV Requirement	Total	Met UV Requirement	Did Not Meet UV Requirement	Total
Adherent to the WHO 6-step protocol	19	2	21	19	6	25
Non-Adherent to the WHO 6-step protocol	0	0	0	0	0	0
Total	19 (90%)	2 (10%)	21 (100%)	19 (76%)	6 (24%)	25 (100%)

**Table 2:** Overview of errors made in hand washing and hand disinfection by all cleanroom operators in the study. The numbers indicate the respective number of cleanroom operators.

	Type of Error	Number of Cleanroom Operators Making an Error	
		Hand Washing	Hand Disinfection
Met requirements	Zero missed areas	17	17
	One minor, missed area (<0.6 cm^2^)	1	2
	Two minor, missed areas (<0.6 cm^2^)	1	0
Did not meet requirements	More than two minor, missed areas (<0.6 cm^2^)	1	0
	One or more major, missed areas (>0.6 cm^2^)	1	6

**Table 3:** Overview of errors related to the time needed to complete hand washing and hand disinfection procedures by all cleanroom operators in the study.

		Hand Washing	Hand Disinfection
		Time (s)	Time (s)
Met requirements	Average	101.6 (n = 18 *)	67.9 (n = 16 *)
	Median	98.9	65.8
	Minimum	56.5	26.8
	Maximum	175.7	108.6
	Standard deviation	26.2	23.8
	Interquartile range	22.8	32.0
Did not meet requirements	Average	123.5 (n = 2)	67.5 (n = 5 **)
	Median	-	65.8
	Minimum	113.8	58.1
	Maximum	133.3	82.4
	Standard deviation	13.8	10.1
	Interquartile range	-	8.6

* Recording of procedure duration was unavailable for three cleanroom operators (in relation to hand disinfection) and one cleanroom operator (in relation to hand washing) that met the requirements. ** Recording of procedure duration was unavailable for one cleanroom operator (in relation to hand disinfection) that did not meet the requirements.

## Data Availability

The raw data can be requested via Fagron Sterile Services Nederland. Personal data are unavailable due to privacy restrictions.
